# Sex differences in gene expression patterns associated with the *APOE4* allele

**DOI:** 10.12688/f1000research.18671.2

**Published:** 2019-07-23

**Authors:** Michelle Hsu, Mehek Dedhia, Wim E Crusio, Anna Delprato

**Affiliations:** 1 Department of Research and Education, BioScience Project, Wakefield, MA, 01880, USA; 2 Institut de Neurosciences Cognitives et Intégratives d'Aquitaine, UMR, CNRS and University of Bordeaux, UMR 5287, Pessac cedex, Aquitaine, 33615, France

**Keywords:** APOE4, Alzheimer's Disease, Systems Genetics

## Abstract

**Background:** The *APOE* gene encodes apolipoprotein ε (ApoE), a protein that associates with lipids to form lipoproteins that package and traffic cholesterol and lipids through the bloodstream. There are at least three different alleles of the *APOE* gene: *APOE2*, *APOE3*, and *APOE4*. The *APOE4* allele increases an individual's risk for developing late-onset Alzheimer disease (AD) in a dose-dependent manner. Sex differences have been reported for AD susceptibility, age of onset, and symptom progression, with females being more affected than males.

**Methods:** In this study, we use a systems biology approach to examine gene expression patterns in the brains of aged female and male individuals who are positive for the *APOE4* allele in order to identify possible sex-related differences that may be relevant to AD.

**Results:** Based on correlation analysis, we identified a large number of genes with an expression pattern similar to that of *APOE* in *APOE4*-positive individuals. The number of these genes was much higher in *APOE4*-positive females than in *APOE4*-positive males, who in turn had more of such genes than *APOE4*-negative control groups. Our findings also indicate a significant sex* genotype interaction for the CNTNAP2 gene, a member of the neurexin family and a significant interaction for brain area*sex* genotype for PSEN2, a risk factor gene for AD.

**Conclusions:** Profiling of these genes using Gene Ontology (GO) term classification, pathway enrichment, and differential expression analysis supports the idea of a transcriptional role of *APOE* with respect to sex differences and AD.

## Introduction

The *APOE* gene encodes the apolipoprotein E protein (ApoE), which associates with lipids to form lipoproteins that package and traffic cholesterol and lipids through the bloodstream ( de Chaves & Narayanaswami, 2008; Eichner *et al.*, 2002; Puglielli *et al.*, 2003). In the central nervous system, ApoE is primarily produced by astrocytes and carries cholesterol to neurons via ApoE receptors of the low-density lipoprotein receptor (LDLR) family ( Getz & Reardon, 2009; Holtzman *et al.*, 2012). There are at least three different alleles of the *APOE* gene: *APOE2*, *APOE3*, and *APOE4*. In each of these variants, there are two distinct amino acid substitutions at positions 112 and 158 that alter the structure and function of the proteins ( Ghebranious *et al.*, 2005; Mahley *et al.*, 2009).

Of the three alleles, *APOE3* is the most common and is believed to have a neutral role in Alzheimer disease (AD), whereas *APOE2*, the least common, is believed to have a protective effect in AD and general cognitive decline. Brains of patients with AD and *APOE2* had fewer fibrillary tangles and lesser amyloid beta deposition. The *APOE2* allele has also been positively associated with better memory performance in a cohort of dementia-free, elderly patients. ( Wu & Zhao, 2016). The *APOE4* polymorphism, which is found in around 20% of the population but 50% of all patients with AD, increases an individual’s risk for developing late-onset AD in a dose-dependent manner ( Ashford, 2004; Raber *et al.*, 2004; van der Flier *et al.*, 2006; Zuo *et al.*, 2006).

Evidence suggests that the *APOE4* allele may contribute to the risk of developing AD due to an increased amount of amyloid plaques in the brain tissue of affected individuals. In turn, a buildup of amyloid plaques may lead to neuronal degeneration and death, resulting in some of the symptoms associated with AD: memory loss, motor function impairment, dementia, and changes in personality ( Barral *et al.*, 2012; Deary *et al.*, 2002; Kim *et al.*, 2009).

Having the *APOE4* mutation does not guarantee that a person will develop AD, indicating that there are other genetic risk factors as well as environment and lifestyle elements that probably contribute to the etiology and progression of the disease.

Differences have been reported between females and males carrying the *APOE4* allele. Women who are positive for *APOE4* have a greater risk of developing AD, show accelerated progression of the disease, and have more severe memory and cognitive decline than men with this allele ( Altmann *et al.*, 2014; Farrer *et al.*, 1997). In a large scale meta-analysis that considered 58,000 research participants, it was also found that females with the *APOE4* allele had a greater risk of developing AD earlier in life than males but that the sex difference was smaller than previously thought ( Neu *et al.*, 2017).

In this study, we examined gene expression patterns associated with aged female and male individuals who are positive for the *APOE4* polymorphism in order to identify possible sex-related differences that may be relevant to the onset and progression of AD. Our hypothesis was that gene expression patterns based on correlation analysis with *APOE* expression in these individuals are different.

## Methods

### Data collection

The RNA sequence data used in this study is derived from the brains of an aged population obtained from the Aging, Dementia and Traumatic Brain Injury Study, accessed through the Allen Brain Atlas ( http://aging.brain-map.org/). To obtain the data, a gene search specifying *APOE* was first performed. *APOE* was then selected and used to extract correlates for each brain region through drop down menus located on the same page. The drop-down menus allowed for the selection of brain region, *APOE4*-positive or -negative data, and sex. Once the selections were made, the “find correlates” button was used to retrieve the data. The datasets consist of gene expression data together with a clinical diagnosis comparison between males and females. The cohorts used in this study are as follows: *APOE4*-positive: females 7, males 13; and *APOE4*-negative: females 31, males 49. In this study only the *APOE4/4* genotype was taken into account. The other possible APOE genotypes were not considered in the analysis because the genotype information was not available.

### *APOE* gene correlates

Genes whose expression correlated with that of the *APOE* gene were collected for the four available brain regions: frontal white matter (FWM) (associated with cognitive function, learning, dementia, and personality changes in AD), hippocampus (HIP), parietal cortex (PCx) (associated with sensory processing, attention, motor function, executive function, and spatial reasoning), and temporal cortex (TCx) (involved in sensory processing as well as declarative and long term memory). Data for each brain region was collected separately for all groups. For information about the RNA-sequence data generation and analysis, see the Quantitative Data Generation white paper in Documentation NA-Seq.

### Correlations evaluation

Correlates to the *APOE* gene were obtained by querying the database and specifying these parameters: brain region, *APOE4* positive and *APOE4* negative, and sex. Correlations equal to or greater than |0.7| were considered in the analysis. The rationale was to investigate genes with a similar expression pattern as *APOE* in these different groups to identify genes specific and common to each group, as well as possibly identify genes related to sex differences. Keyword search of the Database for Annotation, Visualization and Integrated Discovery (DAVID) version 6.8 ( Huang da *et al.*, 2009) table output was used to identify genes associated with biological processes related to *APOE*, sex, and AD. Venny 2.0, an online program that compares lists of items, was used to determine the common and unique genes between groups, sex and brain regions.

### Statistical analysis

Chi square analyses were used to compare the numbers of gene correlates between the *APOE4*-positive and *APOE4*-negative groups and between females and males (Microsoft Office Professional Plus, Excel 2013, Version 15). Gene expression patterns of AD risk factor genes [ *CNTNAP2*, *PSEN2*, *APOE*, *PSEN1*, *APP*, *ADAM10*, and *TREM2*] for *APOE4*-postive and *APOE4*-negative females and males were analyzed by repeated measures ANOVA with genotype and sex as between-subject factors and brain area (FWM, HIP, PCx and TCx) as a within-subject factor. As post hoc test for interactions, Least Squares Means were used (SAS Studio, Release 3.8, Basic Edition). The relationship between CERAD score, Braak stage, and gene expression patterns of AD risk factor genes in APOE4-females and males were analyzed using Pearson product-moment correlations with the HMISC package in R, version 3.6.1. For all statistical analyses α was set to 0.05.

### Gene expression sex differences

The differential search function of the RNA-Seq page of the Allen Brain Database was used to find genes that show enrichment between females and males, and between males and females for each group and each brain region to identify sex related differences in gene expression patterns. Genes with a 1.5-fold difference expression and greater were evaluated.

### Gene Ontology characterization

DAVID was used to obtain functional information based on Gene Ontology (GO) annotations for the gene correlates. Genes associated with a keyword search term related to *APOE*, AD, and sex were identified and noted.

### Pathway enrichment

KEGG pathway enrichment (using DAVID) was used to further characterize the positive and negative *APOE4* gene correlates for each group. Pathways were assessed manually and partitioned based on common themes.

## Results

### The *APOE4* allele alters gene expression patterns

We observed a difference in transcription patterns associated with genes correlating with *APOE* expression in male and female individuals with the *APOE4* allele as compared to *APOE4-*negative individuals. A difference in transcription patterns between *APOE4-*positive females and males was also observed.

As assessed by chi square analyses, the proportions of both positive and negative gene correlates to *APOE* larger than |0.7| were significantly higher in all examined brain regions in both males and females in the *APOE4*-positive than in the *APOE4-*negative groups (Extended data Workbooks 1 and 2; Delprato *et al*., 2019a; Delprato *et al*., 2019b).

Positive correlates: *APOE4-*positive vs *APOE4-*negative groups (all χ ^2 ^have 2 df and p<0.001), females: FWM: χ ^2^=4470.1; HIP: χ ^2^=1285.4; PCx: χ ^2^=4050.4; TCx: χ ^2^=3906.60; males: FWM: χ ^2^=574.8; HIP: χ ^2^=602.8; PCx: χ ^2^=187.4; TCx: χ ^2^=318.5.

Negative correlates: *APOE4-*positive vs *APOE4-*negative groups (all χ ^2 ^have 2 df and p<0.001), females: FWM: χ ^2^=2863.1; HIP: χ ^2^=2654.5; PCx: χ ^2^=2555.1; TCx: χ ^2^=2335.1; males: FWM: χ ^2^=470.8; HIP: χ ^2^=351.4; PCx: χ ^2^=220.3; TCx: χ ^2^=671.1.

In the *APOE4*-positive groups we also observed sex differences. Females carrying the *APOE4*-positive allele had significantly more gene correlates than *APOE4*-positive males. For all brain regions, differences between female and male groups in the numbers of correlates obtained at or above the cutoff value (r=>|0.7|) were assessed with chi square tests (2x2, df=2).

Positive correlates: males *vs* females (all χ ^2 ^have 2 df and p<0.001): FWM: χ ^2^=1926.7; HIP: χ ^2^=237.9; PCx: χ ^2^=2693.4; TCx: χ ^2^=2565.1.

Negative correlates: males *vs* females (all χ ^2 ^have 2 df and p<0.001): FWM: χ ^2^=1051.8; HIP: χ ^2^=1660.9; PCx: χ ^2^=2125.8; TCx: χ ^2^=826.0.

In the *APOE4*-negative groups, sex differences were also observed, but they were less strong than in the *APOE4* positive groups. For the positive correlates, females had significantly more gene correlates than males. However, for the negative correlates, this was reversed for the FWM, whereas no differences were found for the HIP and TCx.

Positive correlates: males vs females (all χ ^2 ^have 2 df and p<0.001 unless indicated otherwise): FWM: χ ^2^=73.6; HIP: χ ^2^=5.3 (0.10>p>0.05); PCx: χ ^2^=6.5 (p<0.05); TCx: χ ^2^=21.8.

Negative correlates: males vs females (all χ ^2 ^have 2 df and p<0.001 unless indicated otherwise): FWM: χ ^2^=150.0; HIP: χ ^2^=3.0 (ns); PCx: χ ^2^=84.1; TCx: χ ^2^=0.9 (ns).

The results indicate that for all brain regions and for both sexes, the number of *APOE* correlates, and therefore gene expression patterns, are significantly different between the groups carrying the *APOE4* allele as compared to *APOE4-*negative individuals and that expression patterns also differ between females and males of the *APOE4-*positive groups.

### Common and unique genes

For the common gene correlates to *APOE* in *APOE4*-positive and *APOE4*-negative individuals, the results for the female and male comparison are as follows: TCx: 47/37 (positive/negative) common genes between females and males, 3263/2532 genes unique to females, 420/577 genes unique to males; HIP 22/16 common genes between females and males, 1164/1984 genes unique to females, 544/323 genes unique to males; FWM 48/22 common gene between females and males, 3465/2335 genes unique to females, 952/818 genes unique to males; PCx 54/42 common gene between females and males, 3432/2605 genes unique to females, 213/175 genes unique to males (Extended data Workbook 3; Delprato *et al*., 2019c).

### GO enrichment

**Figure 1.**
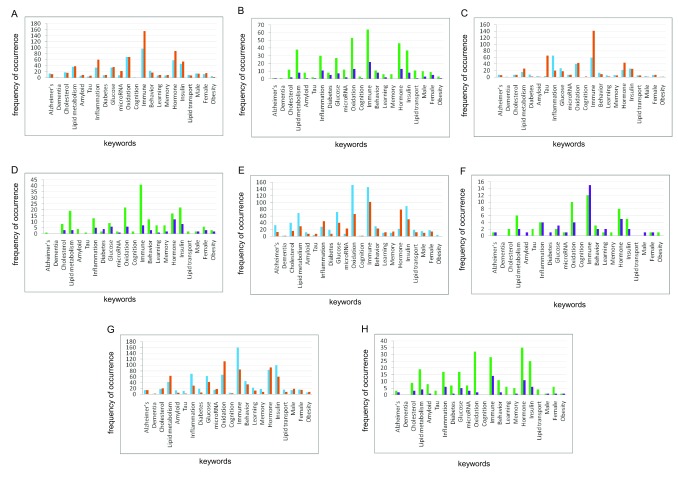
Keyword enrichment. Representative keyword enrichment based on GO Term classification. ( **A**) Females and ( **B**) males, frontal white matter; ( **C**) females and ( **D**) males, hippocampus; ( **E**) females and ( **F**) males, parietal cortex; ( **G**) females and ( **H**) males, temporal cortex. X-axis, keyword categories; Y-axis, frequency of occurrence.

Results from a keyword search of GO terms obtained for positive and negative gene correlates for both the *APOE4*-positive and *APOE4-*negative male and female groups across the four brain regions show the same trend of enrichment ( Figure 1 and Extended data Workbook 4; ( Delprato *et al*., 2019d). Several of the keywords used to analyze the correlates, were associated with a greater number of genes in each brain region and for both positive and negative correlates. The keyword categories are: “immune”, “oxidation”, “inflammation”, “lipid metabolism”, “hormone”, and ”insulin”. Despite the common categories between females and males, there are very few common genes (Extended data Workbook 4; Delprato *et al*., 2019d).

For the keyword categories, there were no common genes between males and females in the FWM correlates, For the other brain regions common genes between males and females are as follows: HIP positive correlates: “inflammation”, *CD4* and *CXCL1*; negative correlates *PPP1CC*; PCx negative correlates “Lipid metabolism” *PTGES3*; “Inflammation” *PTGER4*; “Oxidation” *MTHFD2L*; “Immune”, *FSD1L*, *PTGER4*; “Hormone” *PGRMC2*; TCx negative correlates “Inflammation” *TBC1D23*; “Hormone” *GNRH1*.

Whether either of the female or male groups had more of any one type of gene is not clear, due to the greater number of gene correlates observed for females. In this respect, the female groups generally had more genes in each of these keyword categories overall. The females also have a substantial number of Alzheimer-related genes associated with each brain region (Extended data Workbook 4; Delprato *et al*., 2019d).

### Pathway enrichment

**Figure 2.**
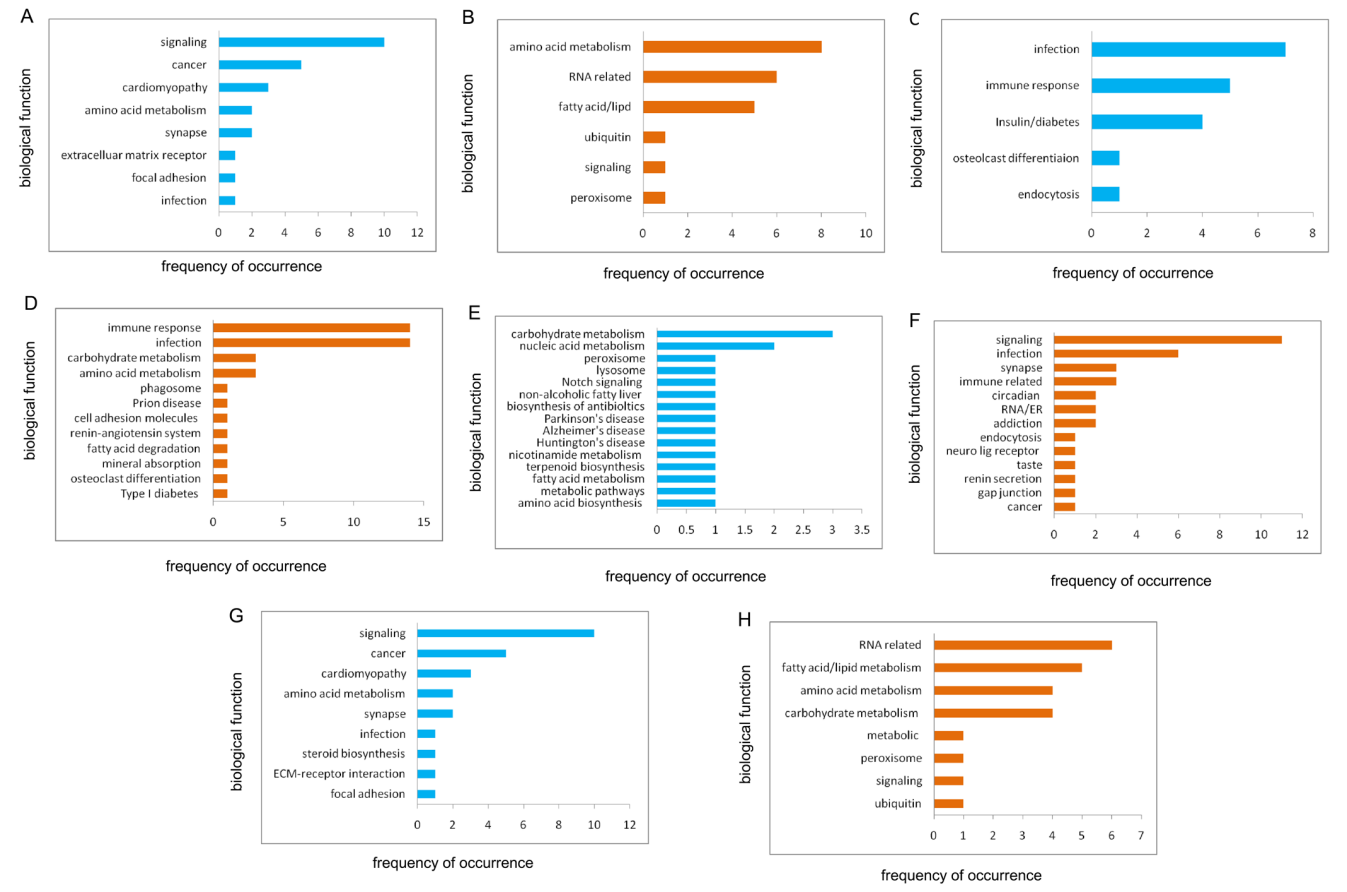
Pathway enrichment. KEGG pathway categories for female gene correlates. ( **A**) Positive correlates and ( **B**) negative correlates, frontal white matter; ( **C**) positive correlates and ( **D**) negative correlates, hippocampus; ( **E**) positive correlates and ( **F**) negative correlates; parietal cortex; ( **G**) positive correlates and ( **G**) negative correlates, temporal cortex. X-axis, frequency of occurrence; Y-axis, biological function.

***Female pathways*.** To gain further insight into the function of the gene correlates, a pathway analysis was performed for each group ( Figure 2 and Extended data Workbook 5; Delprato *et al*., 2019e). For *APOE4-*positive females, the strongest pathway themes, when combined, are associated with various types of signaling cascades, such as MAPK (cell cycle, transcription, stress response), Chemokine (immune response), cAMP (2nd messenger signaling), Jak-STAT (immune function, cell division, apoptosis, TNF (immune system function, inflammation, infection response, apoptosis), Toll-like receptor (immune function, innate immunity), neurotrophin (growth factors, protection, development and function of neurons), VEGF (growth factors, vascularization, cancer), hedgehog (cell differentiation and cancer), infection, immune system processes, amino acid metabolism, lipid metabolism, energy metabolism, RNA related processes, cancer, trafficking and recycling organelles (endosome, lysosome, peroxisome).

The PCx-positive correlates are associated with the AD, Parkinson, and Huntington pathways. There are 16 genes ( *ATP5D*, *ATP6*, *ATP8*, *COX1*, *COX2*, *COX3*, *COX6A2*, *CYTB*, *NDUFA1*, *NDUFA13*, *NDUFA4L2*, *NDUFB7*, *NDUFS6*, *NDUFS7*, *NDUFS8*, *UQCR11*) in common among these pathways and all are related to energy production, mitochondria related-electron transport and oxidative phosphorylation.

Five genes from the HIP negative correlates associated with the Prion disease pathway: *C1QA*, *C7*, *C1QB*, *C6* and *C1QC*; these are all related to the complement pathway of the innate immune system.

***Male pathways*.** For males, the overall number of pathways associated with the *APOE4-*positive gene correlates were much fewer than for the females, and in some cases, such as for the PCx-positive correlates and TCx-negative correlates, there were no pathways identified. The greatest number of pathways for males were obtained for the TCx-positive correlates and these were related to fatty acid metabolism, amino acid biosynthesis, cell attachment/cytoskeleton, neuro-related processes such as GABA axon guidance, viral infection, and immune function. The HIP positive correlates were related to fatty acid metabolism, amino acid metabolism, Lupus, and viral carcinogenesis. For the HIP-negative correlates two pathways were obtained: alcoholism and RNA transport. For the PCx-negative correlates there were four pathways: spliceosome, RNA degradation, NF-κβ signaling and pertussis. For the other brain regions in males there was one pathway for both positive and negative correlates associated with the FWM: Fatty acid/lipid, carbohydrate metabolism, degradation and glycosphingolipid biosynthesis—lacto and neolacto series (Extended data Workbook 5; Delprato *et al*., 2019e).

### Comparison of female and male pathways

Pathways that were exactly the same between female and males were found for the TCx region only, and these are: hsa04510: Focal adhesion, hsa04512: ECM-receptor interaction, hsa05412: Arrhythmogenic right ventricular cardiomyopathy hsa05410: Hypertrophic cardiomyopathy, hsa05414: Dilated cardiomyopathy, hsa04724: Glutamatergic synapse, and hsa00330: Arginine and Proline metabolism.

For the other brain regions, some of the associated pathways were similar in biological function. Common themes between females and males were related to fatty acid, processes, cardiomyopathy, energy metabolism, amino acid metabolism, cell attachment, ECM (extracellular matrix) receptor interaction, glutamatergic synapse, and pathways related to immune function.

Several themes associated with the *APOE4*-positive females stand out as being distinct from male pathways and these are related to cancer, signaling, RNA processes, and a myriad of bacterial, viral, and parasitic infectious disease pathways, which include components of endocytosis, intracellular traffic, and immune system function.

### Differential gene expression between females and males in *APOE4*-positive and *APOE4*-negative groups

**Figure 3.**
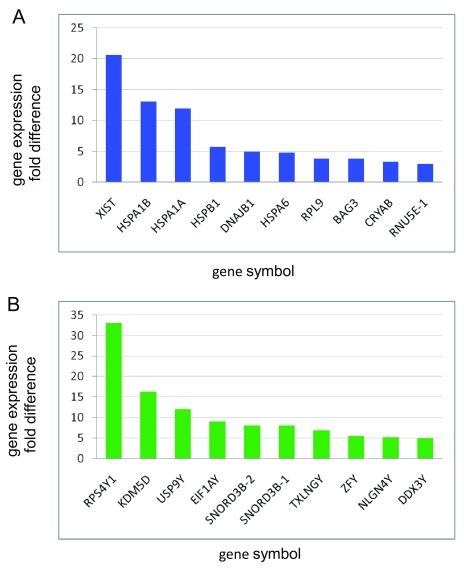
Sex differences in gene expression. ( **A**) Top differentially expressed genes in the frontal white matter of *APOE4*-positive females and ( **B**) representative top genes differentially expressed in *APOE4*-positive males. X-axis, gene symbol; Y-axis, fold difference values.

***Genes expressed higher in females*.** For all groups and for each brain region in the female and male comparison there is one gene, *XIST*, associated with X chromosome inactivation, that is highly expressed and female specific ( Ji *et al.*, 2015). For the *APOE4*-positive group and for each brain region there are several genes that have higher expression values for females over males. Seven of these genes are heat shock proteins, which are involved in the cellular stress response, and chaperones, which assist in protein folding and unfolding: *BAG3*, *DNAJB1*, *CRYAB1*, *HSPA1A*, *HSPA1B*, *HSPA6*, *HSPB1*. The two highest expressed genes aside from *XIST* are *HSPA1A* and *HSPA1B*. The latter two are present in female datasets but not in males. The other top expressing genes in the female and male comparison are *RPL9*, which is down regulated in severe AD ( Kong *et al*. 2009) and *RNU5E-1*, small nuclear RNA ( Figure 3 and Extended data Workbook 6; Delprato *et al*., 2019f).

***Genes expressed higher in males*.** For all groups and brain regions, the same top gene, *RPS4Y1*, which is on the Y chromosome and encodes for the 40S ribosomal subunit, is highly expressed. RPS4Y1 is an RNA binding protein and it is involved in rRNA processing, translation, and protein targeting. Several other genes are expressed at high levels in males. These include *KDM5D* (immune system function, oxidation reduction, T-cell antigen processing and presentation, regulation of androgen receptor signaling pathway), *USP9Y* (ubiquitin/deubiquitination), *SNORD3B-2/1* small nuclear RNAs (RNA biogenesis/transport), *TXLNGY* (syntaxin binding, taxin family), *ZFY* (transcription regulation), *NLGN4Y* (lipid metabolism, synapse assembly, neuron cell-cell adhesion), *DDX3Y* (regulation of gene expression, RNA helicase). With the exception of *SNORD3B-2/1*, all of the other genes are linked to the Y chromosome and are male specific. Other male genes expressed at lower levels but above the cutoff value have diverse functions, including splicing, immune response, and ubiquitination. Based on GO term annotation, none of the top expressing genes in the male and female comparison are heat shock or chaperone proteins but there is one chaperone protein in males just expressed at a 2.0-fold difference, HBB, which is specific for hemoglobin ( Figure 3, Extended data Workbook 6; Delprato *et al*., 2019f).

We also examined the differential gene expression pattern of transcription factors associated with *APOE4*-positive and *APOE4*-negative individuals and we observed distinct expression of transcription factors between these groups and also between females and males (Extended data Workbook 7; Delprato *et al*., 2019g).

There are an increased number of transcription factors expressed in *APOE4*-positive females as compared with *APOE4*-positive males. This is also the case for *APOE4*-positive females as compared to the *APOE4*-negative female group. For males, the *APOE4*-negative and *APOE4*-positive groups have more or less the same set of transcription factors expressed in each brain region with the exception of the FWM in the *APOE4*-negative group.

Transcription factors overexpressed in females as compared to males for *APOE4*-positive groups by brain region are as follows. FWM: *FOS*, *JUN*, *JUNB*, *S100A8*, *A100A9*, *ATF3*, *HSPA1A*, *HSPA1B*, *HMOX1*, *IL1B*, *NPAS4*, *SIK1*, *ZSCAN31*, HIP: *FOS*, *JUNB*, *S100A12*, *NME1-NME2*, *S100A8*, *S100A9*, *ATF3*, *HSPA1A*, *HSPA1B*, *HMOX1*, *NR4A1*, *VEGFA*; PCx: *FOS*, *FOSB*, *JUNB*, *NKX6-2*, *S100A8*, *S100A9*, *ATF3*, *HSPA1A*, *HSPA1B*, *ID3 NR4A1*, *NR4A2*, *SIK1*; TCx: *S100A12*, *S100A9*, *HSPA1A*, *HSPA1B*.

For *APOE4*-negative females, there were no differentially expressed transcription factors observed for the FWM, PCx, and TCx. The exception was for the HIP where *HSPA1A* and *HSPA1B* were expressed above the threshold value of 1.5-fold difference.

In males there are generally the same set of transcription factors for *APOE4*-positive and *APOE4-*negative groups associated with the HIP, PCx, and TCx, and three of these are Y chromosome specific: *KDM5D*, *ZFY,* and *HSFY1*.

An exception was observed for the male *APOE4*-negative group. Associated with the FWM, there were several additional transcription factors expressed above the threshold value ( *FOS*, *CAPN3*, *ENPP2*, *GREM1*, *KDM5D*, *VEGFA*, ZFY and two of these, *FOS* and *VEGFA*, also occur in the female *APOE4*-positive groups ( *FOS*: FWM, HIP, PCx; *VEGFA*: HIP). Otherwise, the expression of transcription factors observed between females and males is distinct.

***Gene expression patterns of AD risk factor genes*.** For *CNTNAP2* we observed significant effects of sex [F(1,72)=6.52, P=0.0128] and brain area [F(3,216)=68.23, P<0.0001] with highest expression in *APOE4*-positive males in the TCx (Extended data Figure 1). Sex interacted significantly with genotype [F(1,72)=5.66, P=0.0316] and brain area [F(3,216)=3.00, P=0.0329].

For *PSEN2* we observed a significant effect for brain area [F(3,213)=20.72, P<0.0001], with a significant brain area*sex*genotype triple interaction [F(3,213)=2.67, P=0.0484]. The highest expression occurred in the TCx for *APOE4*-positive females (Extended data Figure 2).

*APOE* expression was significantly different between brain areas [F(3,207)=49.83, P<0.0001]. There was also a marginally significant sex*genotype interaction [F(1,69)=33.19, P=0.0784] with the highest expression levels in *APOE4*-positive females for the HIP (Extended data Figure 3).

A marginally significant sex*genotype interaction was also observed for *PSEN1* expression levels [F(1,73)=3.26, P=0.0751] and expression differed significantly between brain areas [F(3, 219)=72.11, P<0.0001] with the highest levels observed in *APOE4*-positive females in the FWM (Extended data Figure 4).

For the other risk factor genes, only significant differences between brain areas were obtained: *APP* [F(3,231)=3.92, Pr=0.0105] with highest expression in *APOE4*-positive males for the TCx (Extended data Figure 5), *ADAM10* [F(3,219)=42.01, P <0.0001] with highest expression for *APOE4*-positive males in the FWM (Extended data Figure 6), and *TREM2* [F(3,219)=33.53, P<0.0001] with highest expression for *APOE4*-positive males in the HIP (Extended data Figure 7).

***Relationship between clinico-pathological stages and expression patterns of AD risk factor genes*.** The possible connection between clinico-pathological stages and gene expression patterns for the AD risk factor genes among the *APOE4*-positive and *APOE4*-negative females and males was investigated (Extended data Workbooks 8 to 14, Workbook 1: *CNTNAP2*, Workbook 2: *PSEN2*, Workbook 3: *APOE*, Workbook 4: *PSEN1*, Workbook 5: *APP*, Workbook 6, *ADAM10*, Workbook 7: *TREM2*). The strongest correlations (r |0.7|) occurred between Braak stage and expression of *APOE*: FWM, *APOE4*-positive females: r=0.87, df=5, P=0.054 and *CNTNAP2*, HIP, *APOE4*-positive females, r=0.80, df=5, P=0.057. There were no correlations > |0.7| between CERAD score and the expression of AD risk factor genes. The correlation tables for each gene are included in the extended data workbooks.

## Discussion

The *APOE4* allele is the most significant genetic risk factor for late onset AD ( Corder *et al.*, 1993; Neu *et al.*, 2017; Raber *et al.*, 2004). It is also associated with aging, atherosclerosis, Lewy Body Dementia, and cardiovascular disease ( de Chaves & Narayanaswami 2008; Dickson *et al.*, 2018; Eichner *et al.*, 2002; Riedel *et al.*, 2016). In this study we used a correlation analysis approach to examine gene expression patterns in an aged population consisting of *APOE4*-positive and *APOE4*-negative women and men. The goal of this study was to possibly identify sex-related differences in individuals carrying the *APOE4* allele. This is because many studies have reported differences in the effects of the *APOE4* allele on females particularly, in the context of AD with respect to age of onset and accelerated disease progression associated with dementia and cognitive decline ( Farrer *et al.*, 1997; Podcasy & Epperson, 2016; Riedel *et al.*, 2016; Vest & Pike, 2013). It is important to consider, however, that the male and female differences observed in AD may also be related to the average life span of females who generally live longer than males. Whether this would actually contribute is not directly evident but could be addressed in these types of studies by accounting for the longevity difference in the experimental design.

Our hypothesis was that gene expression patterns in *APOE4*-positive and *APOE4*-negative individuals would differ. We found a large number of genes with the same expression pattern as *APOE* in *APOE4*-positive, but not *APOE4*-negative individuals. This trend was larger in *APOE4*-positive females as compared with *APOE4*-positive males and with *APOE4*-negative control groups.

We did not anticipate that such a large number of gene correlates observed within the significance range would differ between the *APOE4*-positive and *APOE4*-negative groups and also between *APOE4*-positive females and males, even though the expectation had been that there would be differences related to gene function. Despite the differences in the overall numbers of correlates between the groups, the same major categories of genes were observed for both positive and negative correlates as well as for all of the four brain regions considered in this study. The categories of genes relate to immune processes, oxidation, inflammation, lipid metabolism, and hormones.

The results are largely consistent with what is known about *APOE* function. It is interesting, but in retrospect not surprising, that the same gene categories would emerge for all groups despite the substantial differences in the numbers of gene correlates which may be associated with the different biological processes affected by the APOE4 allele.

The results of the pathway analysis were somewhat similar, but some differences were also observed. A large number of pathways were obtained for the female gene correlates, but many fewer for the males, which is not surprising given the larger number of gene correlates for females. Those that could be compared between the two sexes show that there are common themes such as immune function, lipid metabolism, and inflammation. Interestingly, many of the female pathways were related to microbial infection, major signalling circuits, and amino acid metabolism. Several other pathways were concerned with RNA, cancer, and cardiovascular processes. These are very diverse but all relatable to *APOE* function and biological programs.

In the evaluation of genes expressed at higher levels in *APOE4*-positive females than in males, several genes were observed exclusively in females; these were either chaperones that function in protein folding and unfolding or heat shock proteins that play a protective role in cellular stress. The role of these proteins in AD has been investigated ( Campanella *et al.*, 2018; Hamos *et al.*, 1991; Marino Gammazza *et al.*, 2016). In contrast, the genes expressed higher in males than in females were Y-chromosome specific. Distinct patterns of transcription factor expression were also observed between the groups. The transcription factors that are expressed in *APOE4*-positive females are involved in the regulation of a multitude of diverse processes. Several of these have established roles in the regulation of immune system response, inflammation, oxidative stress, aging, and estrogen signaling, which may be of particular relevance to this study (Extended data Workbook 7; Delprato *et al*., 2019g).

We also observed a sex*genotype interaction for the *CNTNAP2* gene. This gene is a member of the neurexin family; is strongly associated with autism spectrum disorders, and has also been linked to AD ( Lazaro *et al*., 2019). In one study, CNTNAP2 was down regulated in the hippocampus of AD patients ( van Abel *et al*., 2012). Interestingly, *CNTNAP2* been has identified as a dyslexia susceptibility gene and several variants are associated with gender specific differences ( Gu *et al.*, 2018). We observed a significant triple interaction of brain area*sex*genotype for *PSEN2*, which is strongly linked to AD and is believed to have a role in APP processing. Disease linked variants of *PSEN2* result in increased production of the longer form of amyloid beta, the major component of amyloid plaques in AD patients (reviewed in Cai *et al.*, 2015).

Others have reported differences in gene expression patterns associated with the *APOE4* allele ( Huang *et al.*, 2017; Lin *et al.*, 2018; Theendakara *et al.*, 2018). The *APOE4* allele has also been shown to alter the transcriptional profile of 857 genes with similar expression patterns to *APOE* in induced neurons and glia ( Lin *et al.*, 2018). The major pathways identified for these genes are consistent with what we observe: lipid processes, immune system, inflammation, energy metabolism, and transcription. There is a growing body of evidence indicating that *APOE* has transcription factor activity. Most of the studies in support of this were done *in vitro* and in mouse models. Further testing in humans is imperative and may provide a clearer understanding of the relationship between *APOE*, aging, and AD.

The results from our study are consistent with known *APOE*-related processes and provide further support for the regulatory role of *APOE* as a transcription factor. That our findings are consistent with other reports that have used different models to study the relationship of *APOE* with AD and aging support the approach that we have taken ( Huang *et al.*, 2017; Lin *et al.*, 2018; Theendakara *et al.*, 2018). We provide further relevance and insight for the differential transcription-related effects of the *APOE4* allele in *APOE4*-positive males and females, which may be related to the overall heightened immune response and increased risk for AD observed in females ( Klein, 2012; Taneja, 2018). The identification of specific genes, gene families, and pathways that are affected by the *APOE4* allele will help to dissect its complex role in diverse but interrelated physiological processes. It would be interesting to compare the results from our study with *APOE4* expression patterns in a younger population to see if these differences between *APOE4*-positive and -negative groups and between females and males are already present and detectable early on or are age dependent.

## Data availability

### Underlying data

Underlying RNA sequence data used in this study was obtained from the Aging, Dementia and Traumatic Brain Injury Study, accessed through the Allen Brain Atlas ( http://aging.brain-map.org/).

### Extended data

Figshare: Workbook 1. Positive and negative gene correlates for *APOE4*+ and *APOE4*- females. Frontal white matter, Hippocampus, Parietal cortex, and Temporal cortex. https://doi.org/10.6084/m9.figshare.7849175 ( Delprato *et al*., 2019a)

Workbook 2. Positive and negative gene correlates for *APOE4*+ and *APOE4*- males. Frontal white matter, Hippocampus, Parietal cortex, and Temporal cortex. https://doi.org/10.6084/m9.figshare.7849190 ( Delprato *et al*., 2019b)

Workbook 3. Common and unique genes for *APOE4*+ and *APOE4*- males and females. Frontal white matter, Hippocampus, Parietal cortex, Temporal cortex https://doi.org/10.6084/m9.figshare.7849196 ( Delprato *et al*., 2019c)

Workbook 4. Keywords for gene correlates *APOE4*+ and *APOE4*- females and males. Frontal white matter, Hippocampus, Parietal cortex, Temporal cortex https://doi.org/10.6084/m9.figshare.7849214 ( Delprato *et al*., 2019d)

Workbook 5. KEGG Pathways for positive and negative correlates associated with *APOE4*+ and females and males. Frontal white matter, Hippocampus, Parietal cortex, Temporal cortex https://doi.org/10.6084/m9.figshare.7849229 ( Delprato *et al*., 2019e)

Workbook 6. Differential gene expression in *APOE4*+ and *APOE4*- females and males. Frontal white matter, Hippocampus, Parietal cortex, Temporal cortex https://doi.org/10.6084/m9.figshare.7849238 ( Delprato *et al*., 2019f)

Workbook 7. Differentially expressed transcription factors in females and males. GO Biological Process. Frontal white matter, Hippocampus, Parietal cortex, Temporal cortex https://doi.org/10.6084/m9.figshare.7849253 ( Delprato *et al.*, 2019g)

Workbook 8. CNTNAP2 gene expression and correlation analysis in *APOE4*-positive and *APOE4*-negative females and males. Frontal white matter, Hippocampus, Parietal cortex, Temporal cortex https://doi.org/10.6084/m9.figshare.8867789 ( Delprato *et al*., 2019h)

Workbook 9. PSEN2 gene expression and correlation analysis in *APOE4*-positive and *APOE4*-negative females and males. Frontal white matter, Hippocampus, Parietal cortex, Temporal cortex https://doi.org/10.6084/m9.figshare.8867774 ( Deplrato *et al*., 2019i) 

Workbook 10. *APOE* gene expression and correlation analysis in *APOE4*-positive and *APOE4*-negative females and males. Frontal white matter, Hippocampus, Parietal cortex, Temporal cortex https://doi.org/10.6084/m9.figshare.8867771 ( Delprato *et al*., 2019j) 

Workbook 11. *PSEN1* gene expression and correlation analysis in *APOE4*-positive and *APOE4*-negative females and males. Frontal white matter, Hippocampus, Parietal cortex, Temporal cortex https://doi.org/10.6084/m9.figshare.8867786 ( Delprato *et al*.,2019k) 

Workbook 12. *APP* gene expression and correlation analysis in *APOE4*-positive and *APOE4*-negative females and males. Frontal white matter, Hippocampus, Parietal cortex, Temporal cortex https://doi.org/10.6084/m9.figshare.8867780 ( Delprato *et al*., 2019l)

Workbook 13. *ADAM10* gene expression and correlation analysis in *APOE4*-positive and *APOE4*-negative females and males. Frontal white matter, Hippocampus, Parietal cortex, Temporal cortex https://doi.org/10.6084/m9.figshare.8867783 ( Delprato *et al*., 2019m)

Workbook 14. *TREM2* gene expression and correlation patterns in *APOE4*-positive and *APOE4*-negative females and males. Frontal white matter, Hippocampus, Parietal cortex, Temporal cortex https://doi.org/10.6084/m9.figshare.8867777 ( Delprato *et al*., 2019n)

Extended data Figure 1. Gene expression graphs for *CNTNAP2*, in *APOE4*-positive and *APOE4*-negative females and males. Frontal white matter, Hippocampus, Parietal cortex, Temporal cortex. A. *APOE4*-positive females, B. *APOE4*-negative females, C. *APOE4*-positive males, D. *APOE4*-negative males Bar graphs represent mean expression +/- SE https://doi.org/10.6084/m9.figshare.8867849 ( Delprato *et al*., 2019o)

Extended data Figure 2. Gene expression graphs for *PSEN2*, in *APOE4*-positive and *APOE4*-negative females and males. Frontal white matter, Hippocampus, Parietal cortex, Temporal cortex. A. *APOE4*-positive females, B. *APOE4*-negative females, C. *APOE4*-positive males, D. *APOE4*-negative males. Bar graphs represent mean expression +/- SE https://doi.org/10.6084/m9.figshare.8867852 ( Delprato *et al*., 2019p)

Extended data Figure 3. Gene expression graphs for *APOE*, in *APOE4*-positive and *APOE4*-negative females and males. Frontal white matter, Hippocampus, Parietal cortex, Temporal cortex. A. *APOE4*-positive females, B. *APOE4*-negative females, C. *APOE4*-positive males, D. *APOE4*-negative males. Bar graphs represent mean expression +/- SE https://doi.org/10.6084/m9.figshare.8867858 ( Delprato *et al*., 2019q)

Extended data Figure 4. Gene expression graphs for *PSEN1*, in *APOE4*-positive and *APOE4*-negative females and males. Frontal white matter, Hippocampus, Parietal cortex, Temporal cortex. A. *APOE4*-positive females, B. *APOE4*-negative females, C. *APOE4*-positive males, D. *APOE4*-negative males. Bar graphs represent mean expression +/- SE https://doi.org/10.6084/m9.figshare.8867840 ( Delprato *et al*., 2019r)

Extended data Figure 5. Gene expression graphs for *APP*, in *APOE4*-positive and *APOE4*-negative females and males. Frontal white matter, Hippocampus, Parietal cortex, Temporal cortex. A. *APOE4*-positive females, B. *APOE4*-negative females, C. *APOE4*-positive males, D. *APOE4*-negative males. Bar graphs represent mean expression +/- SE https://doi.org/10.6084/m9.figshare.8867846 ( Delprato *et al*., 2019s)

Extended data Figure 6. Gene expression graphs for *ADAM10*, in APOE4-positive and *APOE4*-negative females and males. Frontal white matter, Hippocampus, Parietal cortex, Temporal cortex. A. *APOE4*-positive females, B. *APOE4*-negative females, C. *APOE4*-positive males, D. *APOE4*-negative males. Bar graphs represent mean expression +/- SE https://doi.org/10.6084/m9.figshare.8867843 ( Delprato *et al*., 2019t)

Extended data Figure 7. Gene expression graphs for *TREM2*, in *APOE4*-positive and *APOE4*-negative females and males. Frontal white matter, Hippocampus, Parietal cortex, Temporal cortex. A. *APOE4*-positive females, B. *APOE4*-negative females, C. *APOE4*-positive males, D. *APOE4*-negative males. Bar graphs represent mean expression +/- SE https://doi.org/10.6084/m9.figshare.8867855 ( Delprato *et al*., 2019u)

Extended data are available under the terms of the Creative Commons Zero "No rights reserved" data waiver (CC0 1.0 Public domain dedication).
