# Supplementary material for: Sex differences in gene expression patterns associated with the APOE4 allele
Source: F1000Res. 2019 Jul 23;8:387. Originally published 2019 Apr 5. [Version 2] doi: 10.12688/f1000research.18671.2 (PMC6685458; doi:10.12688/f1000research.18671.2)
Supplement: Peer review history

# Peer review: Reviewer response for version 2

**DOI:** 10.5256/f1000research.21927.r51541

**Published:** 2019-08-05

**Authors:** Yu-Wen Alvin Huang, Bo Zhou

**Affiliations:** 1 Department of Molecular and Cellular Physiology, Howard Hughes Medical Institute, Stanford Medical School, Stanford University, Stanford, CA, USA; 2 Institute for Stem Cell Biology and Regenerative Medicine and Department of Pathology, Stanford University Medical School, Stanford, CA, USA

In this revision, the authors have performed additional analyses to address a few concerns as well as interesting questions raised from their original report. This new data, in my view, seriously corroborate their findings for a sex-dependent transcriptional control associated with *APOE* expression. The texts are also modified adequately to convey their points. Hence, I fully approve this revised article with no reservations.

We confirm that we have read this submission and believe that we have an appropriate level of expertise to confirm that it is of an acceptable scientific standard.

# Peer review: Reviewer response for version 1

**DOI:** 10.5256/f1000research.20445.r47508

**Published:** 2019-05-17

**Authors:** Yu-Wen Alvin Huang, Bo Zhou

**Affiliations:** 1 Department of Molecular and Cellular Physiology, Howard Hughes Medical Institute, Stanford Medical School, Stanford University, Stanford, CA, USA; 2 Institute for Stem Cell Biology and Regenerative Medicine and Department of Pathology, Stanford University Medical School, Stanford, CA, USA

The allelic variant of apolipoprotein E (ApoE) gene, *APOE*-e4, is the strongest genetic risk factor for Alzheimer’s disease (AD), with a clearly greater female than male predisposition (reviewed in Belloy et al., 2019 ^1^). Despite the prominence and significance of this *APOE*-by-sex interaction, the underlying mechanism remains to be elucidated. To tackle this critical issue in a comprehensive fashion, Hsu et al. mined the rich data sets of The Aging, Dementia and Traumatic Brain Injury Study, which comprise structured information including transcriptomics, clinical and pathological findings based on 107 aged brains ( http://aging.brain-map.org/). Their functional analyses of genes correlating to *APOE* expression support our current understanding of ApoE's pleiotropy in multiple brain functions and, of note, reveal the female-specific pathways presumably driven by a sex-dependent, transcriptional role of ApoE.

 While their results are informative and very interesting to the AD research community, we would like to suggest the authors to clarify or elaborate a few points, which, in our view, will complement this solid manuscript and better inspire future studies for this underappreciated matter of gender-associated AD risk:     

1. The fundamental approach here is to analyze *APOE* gene correlates – genes whose expression correlated with that of *APOE* – by a built-in function of the aforementioned database online platform, followed by Gene Ontology characterization and pathway enrichment (DAVID). By this approach, the correlation was first computed with normalization to *APOE* transcript level change (=1.0). We recommend that the *APOE* expression pattern to be examined in different groups, including ApoE genotypes, genders, ages and clinico-pathological stages. 
2. To further exploit this valuable database for better insight on the reported results, the information regarding the disease severity - CERAD score and Braak stage – can be used to investigate whether the striking *ApoE4*-positive female pathways operate at an early stage (more likely to be proactive), at a mid-to-late stage (more likely to be reactive) or along the disease progression (like a disease marker). 
3. The authors listed a number of *APOE*-correlated genes that are most differentially expressed in comparison of genders (Figure 3), and most of them represent a well-known cellular function that is important but not conventionally considered to contribute to AD pathogenesis. In the same context of gender-specific ApoE4 effect, do authors mind providing their observation on the expression pattern of other top AD genes (e.g. APP, PSEN1/2, TREM2, etc.) as well as some of risk genes identified in recent GWAS (e.g. Jensen et al., 2019 ^2^)? The results may not be of significant difference but will be of great benefit for understanding how ApoE4 contributes to AD pathogenesis in women.

We confirm that we have read this submission and believe that we have an appropriate level of expertise to confirm that it is of an acceptable scientific standard, however we have significant reservations, as outlined above.

## References

[1] : A Quarter Century of APOE and Alzheimer's Disease: Progress to Date and the Path Forward. *Neuron*.2019;101(5) : 10.1016/j.neuron.2019.01.056 820-838 10.1016/j.neuron.2019.01.056 30844401PMC6407643

[2] : Genome-wide meta-analysis identifies new loci and functional pathways influencing Alzheimer's disease risk. *Nat Genet*.51(3) : 10.1038/s41588-018-0311-9 404-413 10.1038/s41588-018-0311-9 30617256PMC6836675

# Peer review: Reviewer response for version 1

**DOI:** 10.5256/f1000research.20445.r47507

**Published:** 2019-04-30

**Authors:** J.Wesson Ashford

**Affiliations:** 1 Department of Psychiatry and Behavioral Sciences, School of Medicine, Stanford University, Stanford, CA, USA; 2 Director, WRIISD, VA Palo Alto HCS, Livermore, USA

The article by Hsu et al. is an interesting and worthwhile article.

 My 3 problems with such articles are:

- Grouping genotypes as e4 positive or negative – the e4/4 group is as different from the e3/4 group as the e3/4 group is from the e3/3 group, and the e2/4 is very similar to e3/3 – so this is poor data analysis.
- There is an exponential increase of dementia/Alzheimer rate with age, doubling every 5 years, which is rarely taken into account.  Since women live on the average 5 years longer than men, this totally ignored discrepancy can fully account for the male/female difference in Alzheimer incidence, and should definitely be discussed in such studies.  Only precise age adjustments with an exponential term can be correctly interpreted. Most such data sets use 5-year cohorts, which is why this issue is wrongly considered. To really resolve Alzheimer’s disease, more precision of simple epidemiological data is needed.
- A major issue is Leptin, which has a level twice as high in women as men. Leptin is related to energy metabolism and learning, both important issues in AD.

 

 For yet another perspective, see Smith et al., 2019 ^1^.

I confirm that I have read this submission and believe that I have an appropriate level of expertise to confirm that it is of an acceptable scientific standard.

## References

[1] : Putative Survival Advantages in Young Apolipoprotein ɛ4 Carriers are Associated with Increased Neural Stress. *J Alzheimers Dis*.2019;68(3) : 10.3233/JAD-181089 885-923 10.3233/JAD-181089 30814349PMC6484250
